# Estimating Excreted Nutrients to Improve Nutrient Management for Grazing System Dairy Farms

**DOI:** 10.3390/ani13081404

**Published:** 2023-04-19

**Authors:** Sharon R. Aarons, Cameron J. P. Gourley, J. Mark Powell

**Affiliations:** 1 Ellinbank Dairy Centre, Agriculture Victoria Research, 1301 Hazeldean Road, Ellinbank, VIC 3821, Australia; 2 Centre for Agricultural Innovation, School of Agriculture and Food, Faculty of Veterinary and Agricultural Sciences, The University of Melbourne, Melbourne, VIC 3010, Australia; 3 Soil, Water and Nutrients Consulting, Ellinbank, VIC 3820, Australia; cjpgourley@gmail.com; 4 UW-Madison Department of Soil Science, 1525 Observatory Drive, Madison, WI 53706, USA; mark.powell@wisc.edu

**Keywords:** manure, collection, recycling, re-use, nutrient management, nutrient budget, field scale budget

## Abstract

**Simple Summary:**

Improving nutrient use on dairy farms is closely linked to manure deposition, collection and use. Nutrients excreted daily on each one of five visits over a year were calculated based on the energy requirements of grazing lactating cows and information about diets fed to herds on 43 diverse Australian farms. Cows on larger farms, from bigger herds and producing greater milk volumes excreted the most nutrients. Over a year, dairy herds excreted 24, 4, 20, 3, 5 and 3 t of N, P, K, S, Ca, and Mg, respectively, which was up to 66% of most nutrients imported onto farms. Excreted nutrients were allocated to paddocks, dairy shed, yards, feed pads, holding areas and laneways depending on the time the herds spent in these places, with the most deposited in paddocks and the least in dairy sheds. Better collection of nutrients excreted in feed pads and holding areas would reduce nutrient losses by almost a third. The nutrients excreted in paddocks were similar to fertiliser P, and often greater than the fertiliser N, K and Mg applied. However, excretion in paddocks was not uniform, with most nutrients deposited in paddocks closest to the dairy shed. Australian nutrient management tools need to account for excreted nutrients to support identification of on-farm nutrient management opportunities.

**Abstract:**

Improving nutrient management in grazing system dairy farms requires determining nutrient flows through animals, the placement of cows within farms and potential for collection, and the re-use and loss of nutrients. We applied a model incorporating data collected at a range of temporal and spatial scales to quantify nutrient excretion in all locations that lactating herds visited on five days over a year on 43 conventional and organic grazing system dairy farms. The calculated nutrient loads excreted by cows in different places were highly skewed; while N, P and K deposited loads were consistent across the year, S, Ca and Mg loads varied between sampling times and seasons. The greatest mean and range in nutrient loads were deposited in paddocks, with the smallest amounts deposited in dairy sheds. All excreted nutrient loads increased with farm and herd sizes and milk production. Mean daily loads of 112, 15, 85, 11, 22 and 13 kg of N, P, K, S, Ca and Mg were deposited by the herds which, when standardised to a 305-day lactation, amounted to 24, 4, 20, 3, 5 and 3 t excreted annually, respectively. In addition to routine manure collection in dairy sheds, ensuring collection and recycling of nutrients excreted on feed pads and holding areas would decrease potential nutrient losses by 29% on average. Non-collected, recycled nutrients were disproportionately returned to paddocks in which cows spent time overnight, and except for S and Ca, nutrient loading rates were greater than rates applied as fertilisers. These data demonstrate the extent of excreted nutrients in grazing dairy systems and indicate the need to account for these nutrients in nutrient management plans for Australian dairy farms. We propose incorporating excretion data in current budgeting tools using data currently collected on most Australian grazing system dairy farms.

## 1. Introduction

Quantifying nutrient inputs and outputs is a key tool for improving nutrient management and use in dairy production systems worldwide. Globally, dairy production systems have been characterised by nutrient accumulation in response to the intensification of the industry. For instance, farmgate N surpluses on dairy farms ranged from 147 to 609 kg N ha^−1^ for a number of European countries [1,2,3], while P and K surpluses on Portuguese dairy farms varied from 31 to 44 and 52 to 107 kg ha^−1^, respectively [3]. Within grazing systems, median N, P, K and S balances of 193, 26, 74 and 27 kg ha^−1^ (respectively) were reported in Australia [4], and mean annual N and P surpluses of 161 and 28 kg ha^−1^ year^−1^ for New Zealand dairy farms [5].

Nutrient inputs in feed are often the largest contributor to nutrient surpluses in dairy systems [4,6,7,8,9,10,11], which means that nutrient fluxes through the animal (i.e., excreta) are therefore an important aspect of improving nutrient management in dairy systems. In confinement-based systems, most of the manure produced is collected from housing facilities (e.g., barns) for storage and re-application to soil, with the design of collection and treatment facilities dependent on excretion estimation [12,13]. Collection from concreted areas, storage and re-use of excreted nutrients (effluent) are likewise important parts of nutrient management in grazing-based systems, e.g., [14], where the collected nutrients are also used to grow fodder. Furthermore, as grazing-based dairy farms intensify with greater use of imported feed [15], the amount of time cows spend off grazing pasture increases [16], with transfer of excreta from paddocks (fields), where it is mostly immediately recycled, to parts of the farm in which collection and storage systems need to be implemented.

Despite this trend towards greater deposition of manure away from pasture, most animal excreta is still returned directly to paddocks where dairy cows spend the majority of their time, although lactating herds were shown to spend disproportionate amounts of time in certain paddocks [16]. Unless these paddocks are managed to account for increased deposition of excreta, nutrient accumulation is likely to occur. Spatial heterogeneity in soil nutrient levels has been reported on dairy farms [17,18,19,20], where relationships between farm management practices (such as fodder conservation or effluent use) and variability in soil nutrients have been described [17,18,20]. However, variability in the deposition of excreted nutrients has seldom been quantified in grazed dairy systems, which has implications for fertiliser use, animal management, and consequently farm profitability and mitigation of nutrient losses.

The objective of this study was to estimate N, P, K, S, Ca and Mg loads (kg) and loading rates (kg ha^−1^) excreted by lactating dairy cows to estimate nutrient deposition and the extent of nutrient collection and recycling on 43 grazing system dairy farms.

## 2. Materials and Methods

### 2.1. Grazing System Farms

Data were collected at a range of temporal (seasons) and spatial (regional and within farm location) scales from 43 farms located in the eight major dairy-producing regions in Australia; across Temperate, Mediterranean, Sub-tropical and Tropical climatic zones ([16], Table 1). These farms were selected from an initial pool of 124 farms to assess nutrient inputs, outputs and flows associated with systems representative of grazing-based dairy production, as described in more detail by Gourley et al. [4]. The selected farms had a mean stocking rate of 1.7 cows ha^−1^, herd sizes ranging from 51 to 1263, land area from 40 to 460 ha, and included four organic farms. The study farms used a variety of feeding strategies similar to the broader Australian dairy industry [21], ranging from almost complete dependence on grazing pasture to high use of supplementary feeds, with a wide selection of supplementary feeds used [4,10].

### 2.2. Excreted Nutrient Pools

**Figure 1:**
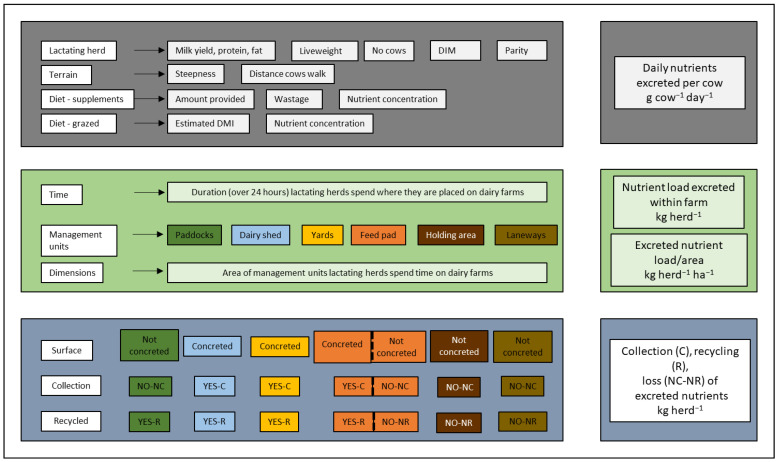
Model consisting of three sub–models showing data used to estimate daily nutrients excreted (g cow^−1^ day^−1^), excreted loads, (kg herd^−1^) and rate (kg herd^−1^ ha^−1^) in management units [16], and potential for collection (C), recycling (R), loss (NC–NR) from management units (kg herd^−1^).

The model (Figure 1) was developed to estimate excreted nutrient pools and consisted of three sub-models that calculated (i) daily nutrient excretion by animals in the lactating herd, (ii) nutrients excreted by the herd in different places on dairy farms, and (iii) the aggregated deposited nutrient loads depending on the potential for recycling or loss based on the infrastructure of each farm.

### 2.3. Nutrient Excretion

Nitrogen, P, K, S, Ca and Mg excreted by the lactating herds were estimated using dietary intake data and feed and milk samples collected at five occasions on each farm [22]. On each visit to a farm, face-to-face interviews were held, during which the farmers provided details about their lactating herds, including milk production, stage of lactation, days in milk, and their dietary intake (pasture and supplements). A modified back calculation method [22,23] based on the metabolisable energy (ME) required for the lactating cows (maintenance, pregnancy, milk production, grazing and activity) and that provided in supplements fed to the animals was used to calculate the ME consumed in pasture. The calculated pasture ME consumed was used in conjunction with pasture sample ME measured in the laboratory to give the pasture DM consumed. Daily nutrient excretion (Table 1) was calculated as the difference between total nutrient intake (pasture and supplements consumed) and nutrients secreted in milk, assuming a net neutral energy balance for each animal in each herd on each day data were collected. This method was devised to enable nutrient excretion estimation for commercial grazing system farms with a variety of lactating breeds, at various stages of lactation and parity, and which use a diversity of dietary strategies. For more details, see Aarons et al. [22].

### 2.4. Deposition of Excreted Nutrients

Excreted nutrients were apportioned to all places on each farm in which the lactating herds were held, based on the time the average cow spent in those places [16]. Briefly, during each on-farm interview, the farmers were asked to describe the places visited by their lactating herds and the time the animals spent in each place in the preceding 24 h. On these farms, the lactating herds were not housed and moved between pasture, paddocks, the dairy shed (milking parlour) and the yards depending on whether they were milked once, twice or three times each day. In addition to paddocks, farmers could have placed their lactating cows on feed pads or in holding areas (loafing or exercise areas). Animals generally travelled between places using laneways (tracks). Most farms had only one lactating herd, but some farms on some occasions had more than one herd that visited different places for different times. Generally, these herds were a smaller group of the main herd held in a separate location for health or other management reasons (Table 1). Based on the information provided by the farmers, the time the average cow in the lactating herd spent in six ‘management’ units (paddocks, dairy shed, yards, laneway, feed pad, holding areas) was calculated. The ‘paddock’ management units were also distinguished as either ‘daytime paddocks’ (paddocks grazed by the cows between milkings during the day) or ‘overnight paddocks’ (the last paddock in which the cows were placed at night and the first paddock they were collected from in the morning). Thus, the six management units were categorised as seven locations (‘daytime’ or ‘overnight’ paddocks, the dairy shed, yards, laneways, feed pads, and holding areas) for excreta deposition analysis. The areas of the locations were calculated using maps created for each farm in ArcGIS 3.3 (ESRI^®^, Redlands, CA, USA) for estimating nutrient loading rates (kg ha^−1^).

### 2.5. Collected and Recycled Pools

The places in which the lactating herds were held on each farm were categorised based on whether they were concreted and excreta could be collected, whether deposited excreta could be directly or indirectly (after collection) recycled, and whether excreta were lost from the system. The calculations do not account for any nutrient losses (e.g., gaseous emissions) associated with deposition or manure management.

### 2.6. Sample Collection

The samples of milk produced and all feed offered (including representative samples of pasture the cows consumed) were collected after each interview. All samples were immediately placed on ice and returned promptly to the laboratory for storage at −20 °C until analysis. Pasture and forage DM were determined after drying at 105 °C for 24 h, and samples were dried at 60 °C for 72 h then ground to less than 2 mm for nutrient analysis. The fodder and milk samples were analysed by Westons Laboratories (Sydney, NSW), for ME, N, P, K, S, Ca and Mg concentrations. Full details of the analytical methodology are given by Aarons et al. [22].

### 2.7. Data and Statistical Analysis

Nutrients (*Ex*, g cow^−1^ day^−1^) excreted by the lactating herd on each farm at each interview were assigned to management units (paddocks, the dairy shed, yards, laneways, feed pads, and holding areas) to give the load (kg) of N, P, K, S, Ca and Mg deposited by the herd on each day (Equation (1)); this resulted in a total of 1464 records (Supplementary Table S1). Unlike the study of Schiavon et al. [24], ammonia volatilisation was not accounted for in this study due to the variation in diets, regional and seasonal climatic conditions which would variously influence losses [25].

(1) $${Load}_{Unit}\left(kg\right)=\frac{\left(Ex\left(g\;{cow}^{-1}\;{day}^{-1}\right)\times {Time}_{pc}\times {Herd}_{Lact}\right)}{1000}$$

where *Load_Unit_* is the amount of a nutrient deposited in one of the management units by the lactating herd on the day of the interview, *Ex* (g cow^−1^ day^−1^) is the amount of a nutrient excreted by each cow on the day of the interview, *Time_pc_* is the percent of the day spent in each management unit, and *Herd_Lact_* is the number of cows in the lactating herd on the interview day.

The management unit data (kg nutrients deposited in each unit per day) were summed to give the total load of excreted nutrients deposited to a management unit by the herd for the day of the interview (*n* = 244; Equation (2)). For the thirteen farms where more than one herd was present, the daily nutrient loads for each herd were averaged (*n* = 211).

(2) $${Load}_{Dy}(kg)=\sum_{24}^{0}{Load}_{Unit}\left(kg\right)$$

where *Load_Dy_* is the total load of nutrient deposited by the lactating herd on the day of the interview.

To calculate annual nutrient loads deposited in each farm by the lactating herds (for comparison with nutrient imports onto farms), daily nutrient loads were multiplied by the number of days designated by the farmer as representative of each quarter. These quarterly loads were summed for the duration of the study and standardised for a 305-day lactation (*n* = 43; Equation (3)).

(3) $${Load}_{Farm–Ann}\left(kg\right)=\frac{(\sum \left({Load}_{Dy}(kg)\times {Time}_{Qtr}(dy)\right))\times 305}{Duration\left(dy\right)}$$

where *Load_Farm–Ann_* is the annual (based on a 305 day lactation) load of a nutrient deposited to all management units on a farm, *Time_Qtr_* is the number of days in each quarter specified by the farmer, and *Duration* is the number of days for the study of each farm.

Daily nutrient loads (kg) were also assigned to locations (‘daytime’ or ‘overnight’ paddocks, the dairy shed, yards, laneways, feed pads, and holding areas) on those farms (i.e., 42 farms) where paddocks could be differentiated based on whether the cows were placed there overnight or only during the day (*n* = 1669; Equation (4)). This analysis was undertaken to investigate herd management decisions affecting excreted nutrient deposition.

(4) $${Load}_{Locn}\left(kg\right)=\frac{Ex\left({g\;cow}^{-1}\;{day}^{-1}\right)\times {Time}_{pc}\times {Herd}_{Lact}}{1000}$$

where *Load_Locn_* is the amount of a nutrient deposited in one of the locations visited by the lactating herd on the day of the interview. 

Annual nutrient loads deposited in these locations were estimated firstly by calculating the loads deposited in each location for each quarter, based on the farmer-designated number of days that are representative of the quarters. The quarterly location nutrient loads were then summed before being standardised for a 305-day lactation (Equation (5)).

(5) $${Load}_{Locn–Ann}\left(kg\right)=\frac{\left(\sum \left({Load}_{Locn}\left(kg\right)\times {Time}_{Qtr}\left(dy\right)\right)\right)\times 305}{Duration\left(dy\right)}$$

where *Load_Locn–Ann_* is the annual (based on a 305 day lactation) load of a nutrient deposited to each location on a farm. 

Daily nutrient loading rates (kg ha^−1^) were then calculated based on the dimensions of each location estimated in ArcGIS (ESRI^®^). To calculate annual loading rates, the nutrient loads deposited in locations visited each day (i.e., the dairy shed, yards, feed pads, and holding areas) were divided by the dimensions (m^2^), multiplied by the number of days in each quarter, summed and standardised for a 305–day lactation (Equation (6)).

(6) $${LoadRate}_{Ann}\left({kg\;ha}^{-1}\right)=\frac{\left(\sum \left((\frac{{Load}_{Locn}\left(kg\right)}{Area\left(m^{2}\right)\times 10,000})\times {Time}_{Qtr}(dy)\right)\right)\times 305}{Duration\left(dy\right)}$$

To estimate loading rates to the day and night paddocks nominated by the farmers at each interview date, the duration of each quarter (as defined by the farmer) was divided by an average rotation length of 21 days before being summed for all quarters and standardised for the lactation (Equation (7)).

(7) $${LoadRate}_{Ann}\left({kgha}^{-1}\right)=\frac{\left(\sum \left(\left(\frac{Load_{Locn}\left(kg\right)}{Area\left(m^{2}\right)\times 10,000}\right)\times \left(\frac{{Time}_{\mathrm{Qtr}}}{21}\right)\left(dy\right)\right)\right)\times 305}{Duration\left(dy\right)}$$

Data manipulation occurred in Access and Excel (Microsoft 2010, Redmond, WA, USA) as well as Genstat 17 (VSN International, 2014, Indore, India), and the latter was used for preliminary exploratory data analyses to understand the structure of the data. Summary statistical analysis (minimum, mean, median, maximum, etc.) revealed that these data were highly skewed and kurtotic, and therefore required logarithmic transformation for further statistical analysis. Residual maximum likelihood (REML) analysis in Genstat 17 was used to analyse the effects of fixed terms (interview dates, seasons regions and management units) on nutrient loads. Genstat REML analysis was also undertaken to analyse the effects of farm characteristics on nutrient loads excreted on farms and in management units on farms. In these analyses, the nutrient deposition data were blocked for the farm (a random term), but not for interview dates, as the herds were different at each interview and therefore the nutrient data could not be considered repeat measurements for each farm. R Studio© Version 1.1.453 was used for graphical presentation of the data. Daily nutrients excreted by herds in management units around the farm were plotted as violin plots to compare the distribution of data based on kernel density distribution. Within each violin, box and whisker plots were drawn, where the box represents the 25th to the 75th percentile, the internal line the median, and the red point the average; the whisker extends to 1.5 times the interquartile range. Axis breaks were created using the ggbreak package [26].

## 3. Results and Discussion

### 3.1. Deposition of Excreted Nutrients

The estimated loads of N, P, K, S, Ca and Mg deposited by lactating herds (Table 2) in places in which they spent time on these farms were highly variable (188% < CV < 204%), as large nutrient loads were excreted in some places in which cows spent considerable time. The excretion load data were also highly positively skewed and kurtotic, with the average load excreted per herd generally about five times greater than the median loads for all nutrients. The range of all data had a minimum of 0.0, as on at least one interview date, lactating cows did not visit (and therefore could not excrete in) a minimum of one of the management units (e.g., the feed pad, holding area or paddocks) present on that farm [16]. This was distinct from farms where these management units did not exist, and consequently, these units were not included in the data analysis for those farms. Significant effects of interview date (0.017 ≤ *p* ≤ 0.022) and season (0.024 ≤ *p* ≤ 0.041) were observed in REML analysis of excreted S, Ca and Mg loads, while excreted N, P and K loads were similar on all visits and in all seasons. These results were unlike previous [27] REML excretion (g cow^−1^ day^−1^) analyses, in which significant interview date and season effects were observed for N, P, S and Mg excretion but not K and Ca. Aarons et al. [16] also reported a significant effect of season was only observed for percent time lactating cows spent in either the dairy shed or yards. All excreted nutrient loads calculated to be deposited by lactating herds were similar, irrespective of the region in which the farms were located.

**Figure 2:**
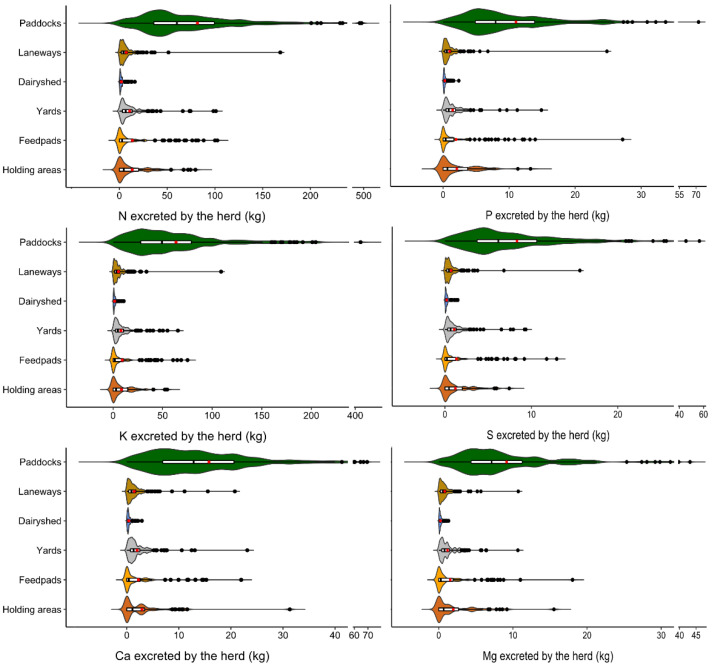
Violin (kernel density) plots of nutrients excreted by lactating herds in paddocks, laneways, dairy sheds, yards, feed pads and holding areas on commercial dairy farms, based on data collected at each of five visits over a year. Box and whisker plots are inside violin plots, with boxes representing the interquartile range (IQR; 25th to 75th percentile), the internal line the median, and the red point the average; the whiskers extend to 1.5 times the IQR.

Significant (*p* < 0.001) differences in loads of excreted nutrients deposited in each management unit (i.e., the paddocks in which cows grazed, feed pads, holding areas, the dairy shed, yards or laneways) were observed (Figure 2). As nutrient deposition was apportioned based on the number of cows being milked at each interview date and the percentage time spent in the management units [16], the differences observed were expected. The biggest range (maximum–minimum) in nutrient loads was deposited in paddocks. For example, on Farm 32, the herd did not visit the paddocks at the end of the study, while on Farm 1, cows spent over 90% of interview days in the paddocks. Despite seasonal differences reported in the time cows spend in the dairy shed [16], the range in nutrient loads deposited in that management unit was the smallest, as generally, per cow time spent milking was relatively constant. Deposition of nutrients in laneways also varied widely due to the range in distances walked. The mean distances walked between paddocks and the dairy shed on these farms ranged from 0.22 to 1.72 km, with some herds on some interview dates walking as much as 2.68 km one way. Thus, after paddocks, the largest range in N, K and S occurred in laneways. The next biggest range was in feed pads for P and Mg, and in holding areas for Ca, potentially associated with the use of these areas at times during the year when supplementary feeds had greater contents of these nutrients. For instance P, Ca and Mg concentrations in minerals were the highest for feeds supplied (dietary feed lime and a commercial mineral mix) when the animals used feed pads [27].

When the data were analysed for a main effect of management unit crossed with interview date, season, or region significant management unit (*p* < 0.001) and interview date (*p* < 0.044) or season (*p* < 0.009), effects were observed for all excreted nutrient loads except for N, for which the loads were similar irrespective of season. This result may possibly be due to the strong relationship between dietary N and milk production, leading farmers’ selection of diets to ensure a consistent supply of N. Region effects were never significant. Although management unit and region interactions were significant (*p* < 0.001), management unit and interview date and management unit and season interactions were not observed.

The farm characteristics of farm size, herd size, total, per cow and per hectare milk production each had positive effects on all excreted nutrient loads, with highly significant (*p* < 0.001) effects observed for farm size, herd size, and total milk production (Table 3). In contrast, highly significant effects between mean daily nutrient excretion (g cow^−1^ day^−1^) and herd size and total milk production were only observed for N and S [22]. The effects of per ha milk production on excreted nutrients were least strong for Ca (*p* = 0.034), with more significant effects observed for all other nutrients (0.002 ≤ *p* ≤ 0.005); however, only N, P and S daily excretion had been related to per ha milk produced on these farms [22]. Nitrogen, P and Mg loads were strongly significantly (0.001 ≥ *p* ≤ 0.003) related to per cow milk production, as was daily excretion [22], while significant but weaker effects (0.013 < *p* < 0.032) were observed for K, S and Ca. Stocking rate effects on N, K and S loads (0.011 < *p* < 0.023) excreted by herds on these farms were observed too, while only a potential relationship was observed for P and Mg; this is in contrast to daily excretion, for which positive effects between N (*p* = 0.004) and S (*p* < 0.001) and a negative effect for Ca (*p* = 0.046) were observed. Phosphorus loads excreted by herds on farms only appeared (*p* = 0.051) to increase as the percentage of feed ME imported onto the farm increased, although increases in daily per cow P and Mg excretion and decreases in K excretion were previously reported [22].

The relationships described above are based on data collected at five quarterly interviews [16,22] rather than annual farm data [4]. Positive relationships between farm scale nutrient balance and milk production could explain relationships between excreted nutrients and milk production observed in the current study. On the other hand, excreted nutrient loads were weakly related to stocking rates in this study compared to previously reported strong positive relationships between farm stocking rate and N, P, K and S balances [4]. Ledgard et al. [28] also noted that stocking rate is not a strong determinant of excreted N and therefore N losses, which they attributed to between-farm variations in N intake and cow production. In this study, the data were split by farm in the statistical analysis to account for farm-specific effects on the data. However, the use of all cows (lactating, heifers and dry cattle) in the calculation of farm-scale stocking rate could explain differences in relationships observed between this and the Gourley et al [4] study. As for the positive relationships between farm land area and per hectare nutrient balances [4], similar strong relationships between excreted nutrient loads and land area were observed in this study.

Analysis of nutrients excreted by herds in each management unit showed excretion in the dairy shed and yards (*p* < 0.001) was always positively affected by land area, cow numbers and total milk production. Likewise, nutrient excretion in paddocks and laneways showed very strong effects of herd size (*p* < 0.001), total milk produced (*p* < 0.001; *p* ≤ 0.003, respectively) and farm area (*p* ≤ 0.002; 0.001 < *p* ≤ 0.006, respectively). Generally, poorer effects were observed for nutrient loads excreted in feed pads and holding areas; this is most likely due to the smaller amount of data for these farm characteristics. Nutrient excretion on feed pads did not appear to be related to farm size, as feed pads occurred on farms of all sizes (40 to 430 ha). Except for Ca, all nutrients excreted in feed pads were positively affected by the number of cows (0.005 ≤ *p* ≤ 0.033) and milk production (0.003 ≤ *p* ≤ 0.026), perhaps an indication of the greater use of this management unit on farms milking more cows. Potassium and S loads excreted in holding areas (*p* = 0.035, *p* = 0.03, respectively) were more strongly influenced by farm size than the other nutrients, but no significant relationships between milk production or herd size and any nutrients excreted in holding areas were observed.

Except for Ca (0.022 ≤ *p* ≤ 0.045), the other nutrients excreted in dairy sheds, yards, paddocks and laneways were strongly (0.002 ≤ *p* ≤ 0.008) influenced by per hectare milk production, and less so (0.022 ≤ *p* ≤ 0.048) for nutrient loads excreted in feed pads. No effects of per hectare milk production on nutrients excreted in holding areas were observed. Again, except for Ca, strong effects of per cow milk production were observed for the other nutrients excreted in holding areas and yards. Excreted P (0.005 ≤ *p* ≤ 0.006) and Mg (0.006 ≤ *p* ≤ 0.010) in the laneways and dairy sheds showed strong relationships with per cow milk production, as did N excretion in feed pads (*p* = 0.007).

The strongest effects of farm stocking rate on excreted nutrients occurred in paddocks (0.002 ≤ *p* ≤ 0.048), followed by dairy sheds (0.006 ≤ *p* ≤ 0.032) and laneways (0.013 ≤ *p* ≤ *0.046*). Only excreted N (*p* = 0.041), K (*p* = 0.027) and S (*p* = 0.022) increased in the yards with stocking rate, while the potential for (at least) K and S excreted loads to increase with the stocking rate in feed pads was observed. Stocking rate did not seem to influence nutrients deposited in holding areas. By contrast, however, deposition of all nutrients in holding areas increased (0.005 ≤ *p* ≤ 0.019) with the importation of supplementary ME (as a percentage of dietary intake), while only excreted P (*p* = 0.036) increased in the yards.

The influence of farm characteristics (based on annual whole-of-farm data) on daily excretion estimates indicates that larger farms (ha), bigger herds, and larger milk production can be associated with greater excreted herd nutrients, particularly in dairy sheds, yards, laneways and paddocks. It is important to note that these observed relationships are likely to be most influenced by the time the cows spent in places on the farms, acknowledging potential covariate relationships between say farm and herd size and milk produced. Future research examining farm and herd factors influencing excreted nutrient loads would greatly assist improvements in excreta management in these systems. For instance, herd size and total milk produced were strongly related to excreted nutrients deposited in feed pads, wherein farms with feed pads on average appeared to have larger herds, greater milk production and more imported supplementary feeds. Nutrients deposited in holding areas appeared to be strongly influenced by the percentage of feed ME imported, as animals were typically held for extended periods in these areas for supplementary feed as well as other reasons. Excretion in feed pads and holding areas increased with per cow milk production, while nutrients excreted in paddocks was related to per ha milk produced. Of all the minerals, excreted Ca appeared the least responsive to farm characteristics, presumably because of greater homeostatic regulation by the animals, but also due to the less frequent dietary Ca supplementation in these herds [22,29].

When the nutrient loads excreted in each management unit visited over the 24 h were summed to give the total nutrients excreted for the day of the interview, the daily loads excreted by the herd were less variable (76% < CV < 89%; Table 4) than the loads excreted in different places on farms (Table 2). Median loads of 82, 11, 64, 8, 18 and 10 kg of N, P, K, S, Ca and Mg were deposited by the herds on these farms over each day. The mean loads were about 130% greater than the median, and maximum daily loads of almost an order of magnitude greater were observed on some interview dates. Significant differences were observed in mean daily P (*p* < 0.001), S (*p* < 0.001), Ca (*p* = 0.001) and Mg (*p* = 0.002) loads excreted on the interview dates, but not N or K. The greatest excretion was recorded at the fourth (October/November 2008) interview, and the least at the previous quarterly interview (July/August 2008) for S, Ca, and Mg, while P was lowest at the last interview (January/February 2009). Similarly, significant seasonal differences were observed for N (*p* = 0.019), P and S (*p* < 0.001), Ca (*p* = 0.001) and Mg (*p* = 0.004). The greatest loads were excreted in spring for these nutrients, but the smallest amounts excreted were either in autumn (S, Mg), summer (P, N) or winter (Ca). In this study, most herds calved in spring (October/November 2008), and their increased milk production would be associated with greater feed intake and a corresponding increase in excreted nutrients [12,13].

Using the daily herd excretion data, accounting for the duration of each quarter as specified by the farmers and standardising to a 305–day lactation, the median loads excreted by these herds amounted to 24, 4, 20, 3, 5 and 3 t N, P, K, S, Ca and Mg, respectively (Table 5). When compared with the total nutrients brought onto these grazing system dairy farms over the year of the study [30], the excreted nutrients were on average 66% of N, P and Mg, and 48%, and 33% of S and Ca imports, respectively. Excreted K was 1.5 times that imported onto farms that year, indicating that K had accumulated on these farms in previous years.

Based on data reported by Gustafson et al. [31] and accounting for depositions to pasture [32], the internal flows in manure and urine of N, P, K, S, Ca and Mg averaged 60, 71, 80, 95, 73 and 99%, respectively, of the external flows of a conventional and an organic Swedish dairy farm over three years. On the organic Australian dairy farms, excreted P and Ca made up a much smaller percentage of total imports (21 and 8%, respectively), compared with the other nutrients. Excreted N and Mg as a proportion of total imports on organic farms were similar to those on conventional farms (Table 5). However, K in excreta was marginally greater, while excreted S was a much greater proportion of total imports on organic farms compared with conventional farms. Fertiliser P use on these organic farms was restricted to rock phosphate, which has low solubility and consequently would not be readily taken up by the pasture, thereby limiting the potential recycling of P in excreta. Sulphate of potash (K_2_SO_4_) was frequently used [4] on the organic farms, with its solubility likely to contribute to high recycling of K and S in excreta.

These data demonstrate the importance of imported nutrients, not just for farm-scale budgets, but for within-farm nutrient flows and cycling as influenced by excreted nutrients. Similarly, Kobayashi et al. [33] reported nutrient flows in animal excreta and sawdust bedding that, averaged over 5 years, were 60% of fertiliser, feed and sawdust N and P imports and 1.2 times the K imports onto their study farm. By contrast, animal excreta was 2.8, 1.6 and 7.8 times greater than chemical fertilizer imports of N, P and K onto the farm. Thus, within grazing systems, excreted nutrients are likely to constitute a significant part of nutrient flows as well as potential losses.

### 3.2. Nutrient Recovery and Re–Use

Nutrients deposited in excreta on grazing system dairy farms can be assigned to different pools based on the potential for the nutrients to be captured/collected for re–use, directly recycled on paddocks, or lost from the production system. On most dairy farms, excreted nutrients are managed for re-use after collection in the dairy shed, yards, and concreted infrastructure (e.g., housing, feed pads); in our study, they constituted on average only about 9% of the uncollected/directly recycled nutrient pool (i.e., deposited on grazed paddocks). On average, 1.3 times the collected nutrients were lost from the system on laneways and in non–concreted feed pads and holding areas (Table 6). Nutrients lost on laneways are not easily recovered, apart from by minimising the time for which cows wait in these places. However, uncaptured losses from feed pads and holding areas are unnecessary. Seventeen of these 43 farms had feed pads and/or holding areas from which excreted nutrients were not collected for re–use, with mean daily loads of N, P, K, S, Ca and Mg deposited amounting to 13.2, 2.2, 9, 1.3, 2.6 and 1.9 kg, respectively, or 4, 0.7, 2.8, 0.4, 0.9 and 0.6 t, respectively over a 305-day lactation. Nutrients in the uncollected/lost pool would on average decline by between 22% to 34% by ensuring the collection and recycling of nutrients deposited on feed pads and holding areas (Table 7). Furthermore, decreases in environmental impact associated with greater capture of excreted nutrients, particularly N and P, are difficult to estimate for these farms due to differences in edaphic factors as well as farm and manure management [34].

Nutrients retained on farms, when collection of excreta from feed pads and holding areas is improved, are generally a small proportion of the nutrients deposited and recycled in grazing paddocks. However, excreta loads are not expected to be uniformly deposited on paddocks around farms as a result of where and for how long farmers place their herds. Estimated mean nutrient loads deposited to paddocks where the cows spent the night were about one and a half times greater than that returned to paddocks visited between the morning and evening milking (Table 8). As paddocks in which the animals spent time overnight are typically selected to assist with managing cows for the morning milking, they were reported to be significantly closer to the dairy shed than the paddocks visited during the day [16]. The accumulation of nutrients around the dairy sheds, due to the greater nutrient loads deposited overnight in paddocks, is further exacerbated by excreta deposition in feed pads and holding areas which were reported to be less than 100 m from the dairy shed on average [16] (see Table 8). Moreover, nutrients collected from the dairy shed and yards are typically applied as effluent to the paddocks closest to the dairy shed [18]. Thus, these calculated excreted nutrient data support the accumulation of nutrients reported near dairy sheds and milking parlours on grazing system farms [18,19,20].

### 3.3. Nutrient Loading Rates

While mean nutrient returns to the paddocks in which cows were placed overnight or during the day were greater than other locations on these farms, the loading rates (kg ha^−1^) were considerably less than that for feed pads and were similar to deposition in holding areas, as the latter were frequently paddocks in which the cows were kept for extended periods. Mean daily loading rates of excreted N, P, K and Mg to paddocks in which cows were placed overnight were greater than that applied as fertilisers, with at least three times as much K and Mg in deposited excreta compared with mean fertiliser applications on these farms (Table 9). Less N, P and S were deposited by the lactating herd in paddocks during the day when compared with the application of these nutrients in fertilisers, while K and Mg were deposited in these paddocks at a minimum of twice the loading rate of fertiliser. Calcium was the only nutrient deposited at rates well below that applied in fertiliser, most likely due to the limited use of dietary Ca and the application of fertiliser lime on these farms. However, when the influence of high Ca fertiliser application was accounted for (i.e., median data), the difference between excreted and fertiliser Ca was less.

## 4. Nutrient Management Recommendations for These Grazing Systems

Losses from uncollected/lost nutrient pools can lead to water and air quality degradation and greenhouse gas emissions [35], while nutrient accumulation associated with animal placement can result in imbalances in soil and pasture nutrients, thereby causing animal metabolic issues [36,37]. Accurate estimation of nutrient losses is not possible for the diversity of farm systems and environments in this study. However, selection of mitigation strategies, e.g., [38], to minimise losses and contamination of the environment will benefit from quantification of within-farm nutrient flows; these will also assist in the management of nutrients for production benefits.

Nutrient management tools for the Australian dairy industry are based on farm gate balances used to determine fertiliser application rates for maintaining fertility or building soil nutrient levels [39,40]. These budgets do not account for all within–farm flows of excreted nutrients, only estimating collected nutrients (effluent) as well as nutrient losses in laneways. The grazing system farm data in this study show that collected and recycled nutrients as well as lost nutrients make up less than 20% of excreted nutrients, and therefore a significant proportion of deposited nutrients are not taken into consideration in these tools.

Furthermore, our research indicates a need to account for the temporal and spatial variability in inputs to paddocks. We propose that a number of ‘management units’ should be considered when developing nutrient accounting tools for grazing systems. Paddocks in which cows are placed overnight need to be distinguished from paddocks used during the daytime, particularly if the former are not part of the grazing rotation used for the day paddocks. Holding areas, which in this study were not concreted for any of the farms nationally, also need to be identified, as nutrient accumulation and loss in these areas would be expected to be high. Feed pads could be considered as similar to yards if these are concreted and excreta are routinely collected. On the other hand, in many instances, excreta deposited in earthen feed pads was infrequently scraped and stockpiled or not collected. Nutrient losses from these areas could be different and greater than those areas in which feed pads are concreted. As dependence on importation of supplementary feed grows [41] and farms expand, feed pads are increasingly used and need to be appropriately managed to minimise nutrient losses. Further, our results suggest that larger farms with greater milk production are likely to have bigger excreted nutrient loads in non–concreted or holding areas requiring management.

The estimation of on–farm effluent nutrients in the current dairy nutrient management planning tool is based on averages from historical data or require the farmer to collect representative samples from their effluent ponds for analysis. Similarly, calculation of nutrient losses is based on generalisations of travel distances and waiting times for their herd. The approach used in this research estimated nutrient loads deposited based on nutrient excretion rates calculated from milk production, herd diet and metabolic requirements [27], as well as from determining actual times animals spent in locations [16,42]. While accurate estimate of pasture intake is currently a limitation, this method offers promise for more accurately apportioning nutrients to the different places in which cows spend time on grazing system farms.

Much of the data that are required to estimate nutrient inputs (and outputs) at a field scale are currently collected on modern dairy farms, as they have increasingly adopted sensor technologies. Thus, many farms use recognition data in animal houses to target supplementary feeding of lactating dairy cows; this may be through the use of electronic collection of data on liveweight, stage of lactation and milk yield [41]. These data can therefore be used to compute herd nutrient excretion rates. By combining the time the animals spend in the different ‘field types’, either manually or (as the technology and data analysis techniques improve) by using global positioning technologies [43], the deposition of excreted nutrients around farms can be estimated. Thus, the incorporation of dietary intake data and cow location information into spatial tools that map farm field types will allow for more precise spatial accounting of excreted nutrients.

In these grazing systems, other nutrient flows such as fodder removal by grazing or mechanical harvesting (i.e., for silage or hay), fertiliser inputs, nitrogen fixation, irrigation and effluent application should be measured. Of these flows, fertiliser inputs are typically the least spatially variable, while N fixation will depend on pasture legume content. By accounting for all within-farm nutrient flows, fertiliser nutrients can be more precisely targeted, while areas within farms in which the risk of nutrient accumulation and potential loss is greatest (and therefore where mitigation is most required) can be more readily identified.

## 5. Conclusions

Lactating herds on grazing system dairy farms excrete large nutrient loads which are influenced by farm productivity metrics such as milk yield, with excreta deposition in places such as holding areas related to importation of supplementary feed onto farms. Nutrients excreted by these herds are a significant proportion of total nutrient imports onto these farms, with deposition on grazed paddocks at least equal to or greater than nutrients applied as fertiliser. Improving recovery and re–use of nutrients excreted in but not currently collected from feed pads and holding areas will reduce nutrient losses from these farms. Current nutrient management tools need to incorporate estimates of non-uniform animal excreta deposition in paddocks as well as excreta collected for re–use from feed pads and holding areas.

As the Australian dairy industry intensifies and grazing system farms increase nutrient inputs, particularly in the form of supplementary feeds, the pool of potentially collectable nutrients will grow. The potential exists to estimate the excreted nutrients deposited for collection or in paddocks to improve both the management of excreta and fertiliser nutrient application, through the use of electronic herd production and intake information currently available on farms in conjunction with geospatial technologies.

## Figures and Tables

**Table 1:** Mean (range) characteristics of 43 grazing system dairy farms as well as data collected on five interview dates for lactating cows including herd sizes, diet metabolisable energy, and nutrients excreted.

**Farm Characteristics Data**	
Farm area ^a^ (ha)	194 (40–460)
Herd size ^b^	296 (51–1263)
Stocking rate ^c^ (cows ha^–1^)	1.7 (0.4–3.7)
Total milk produced ^d^ (kL)	2229 (373–11247)
Feed ME imported ^e^ (%)	33 (0–66)
Number of farms with	
More than 1 lactating herd ^f^	13
Feed pad(s)	20
Holding area(s)	11
Feed pad(s) and holding area(s)	7
**Interview Date (Quarterly) Data ^g^**	
Lactating herd size ^h^	264 (30–1350)
Total ME ^i^ (MJ cow day^–1^)	195 (116–289)
Supplement ME fed (MJ cow day^–1^)	104 (13–251)
Calculated pasture ME (MJ cow day^–1^)	98 (0.4–236)
Excreted nutrients ^j^ (g cow day^–1^)	
N	433 (199–793)
P	61 (20–132)
K	341 (140–671)
S	44 (19–101)
Ca	92 (10–210)
Mg	53 (21–274)

^a^ Refers to the land area that the lactating herd regularly visits [see 4 for further details], ^b^ Total number of lactating, dry and springing cows averaged from data collected at five quarterly on–farm interviews, ^c^ Stocking rate applied to the land that the lactating herd regularly contacts as distinct from the home farm area or all land that the farmer uses as part of their production system [4], ^d^ Milk produced on each farm for the study year, ^e^ Feed metabolisable energy requirements (ME) as a percentage of total ME requirements that is imported on to farms, ^f^ Five of the 13 farms had more than one lactating herds on at least three interview dates, ^g^ Data collected at each of five interview dates on each farm and summarized, ^h^ Number of cows in the lactating herds on all farms at all interviews, ^i^ Metabolisable energy. Pasture ME calculated after subtracting dietary ME in supplements fed from the total ME required for milk production, maintenance, pregnancy, activity and grazing [22], ^j^ Excreted nutrients calculated after subtracting dietary nutrient intake (supplements and pasture) from nutrients secreted in milk.

**Table 2:** Summary statistics for the estimated daily load (kg herd^−1^) of excreted nutrients (N, P, K, S, Ca, Mg) deposited by lactating herds on 43 grazing system dairy farms, based on the time the cows spent in all places ^a^ visited over the 24 h preceding each of the five interview dates.

	Daily Excreted Nutrient Load Deposited (kg Herd^−1^)					
	N	P	K	S	Ca	Mg
No. of records	1162	1162	1162	1162	1152	1152
Minimum	0	0	0	0	0	0
Mean	23.5	3.2	17.9	2.4	4.5	2.7
Median	5.04	0.71	3.93	0.51	1.05	0.61
Maximum	495	72	409	57	69	43
Std Dev	47.66	6.35	35.83	4.85	8.74	5.01
CV ^b^ (%)	203	198	200	204	194	188
Skew ^c^	4.42	4.15	4.29	4.42	3.63	3.62
Kurt ^d^	27.54	24.61	27.27	28.57	16.59	16.99

^a^ On these farms, cows could have visited a combination of paddocks in which they grazed, feed pads, and/or holding areas, and used laneways to walk between these places and the dairy shed and yards for milking, ^b^ CV coefficient of variation, ^c^ skewness; standard error of skewness = 0.0718, ^d^ kurtosis; standard error of kurtosis = 0.143.

**Table 3:** *F–prob*^a^ estimates for positive effects of farm characteristics on nutrient loads excreted on 43 grazing system farms, and for positive effects of farm characteristics on daily nutrient loads excreted in management units (dairy sheds, feed pads, holding areas, laneways, paddocks, yards) on these farms.

		N	P	K	S	Ca	Mg
Farm area ^b^ (ha)		<0.001	<0.001	<0.001	<0.001	<0.001	<0.001
	Dairy shed	<0.001	<0.001	<0.001	<0.001	<0.001	<0.001
	Feed pads	ns	ns	ns	ns	ns	ns
	Holding areas	0.046	0.048	0.035	0.03	ns	0.055
	Laneways	0.001	0.003	0.003	0.004	0.006	0.005
	Paddocks	<0.001	<0.001	0.001	0.001	0.002	<0.001
	Yards	<0.001	<0.001	<0.001	<0.001	<0.001	0.001
Herd size ^c^		<0.001	<0.001	<0.001	<0.001	<0.001	<0.001
	Dairy shed	<0.001	<0.001	<0.001	<0.001	<0.001	<0.001
	Feed pads	0.013	0.033	0.017	0.017	0.056	0.005
	Holding areas	ns	ns	ns	ns	ns	ns
	Laneways	<0.001	<0.001	<0.001	<0.001	<0.001	<0.001
	Paddocks	<0.001	<0.001	<0.001	<0.001	<0.001	<0.001
	Yards	<0.001	<0.001	<0.001	<0.001	<0.001	<0.001
Stocking rate ^d^ (cows ha^−1^)		0.023	0.062	0.014	0.011	ns	0.058
	Dairy shed	0.015	0.032	0.009	0.006	ns	0.029
	Feed pads	0.072	Ns	0.051	0.056	ns	0.062
	Holding areas	ns	ns	ns	ns	ns	ns
	Laneways	0.024	0.054	0.022	0.013	ns	0.046
	Paddocks	0.004	0.009	0.004	0.002	0.048	0.007
	Yards	0.041	ns	0.027	0.022	ns	ns
Total milk produced ^e^ (L)		<0.001	<0.001	<0.001	<0.001	<0.001	<0.001
	Dairy shed	<0.001	<0.001	<0.001	<0.001	<0.001	<0.001
	Feed pads	0.009	0.026	0.013	0.012	0.046	0.003
	Holding areas	ns	0.071	0.053	0.061	ns	ns
	Laneways	<0.001	0.003	<0.001	<0.001	<0.001	<0.001
	Paddocks	<0.001	<0.001	<0.001	<0.001	<0.001	<0.001
	Yards	<0.001	<0.001	<0.001	<0.001	<0.001	<0.001
Per cow milk produced ^f^ (L cow^−1^)		0.003	<0.001	0.021	0.013	0.032	0.001
	Dairy shed	0.02	0.006	ns	0.065	ns	0.01
	Feed pads	0.007	0.016	0.025	0.01	0.064	0.012
	Holding areas	<0.001	<0.001	<0.001	0.001	0.012	0.001
	Laneways	0.01	0.005	0.06	0.037	0.074	0.006
	Paddocks	ns	ns	ns	ns	ns	ns
	Yards	0.001	<0.001	0.006	0.005	0.01	<0.001
Per ha milk produced ^g^ (L ha^−1^)		0.002	0.003	0.002	0.002	0.034	0.005
	Dairy shed	0.002	0.003	0.004	0.002	0.038	0.004
	Feed pads	0.031	0.048	0.03	0.027	ns	0.022
	Holding areas	ns	ns	ns	ns	ns	ns
	Laneways	0.003	0.005	0.006	0.003	0.045	0.005
	Paddocks	0.003	0.003	0.008	0.003	0.061	0.004
	Yards	0.002	0.003	0.002	0.002	0.022	0.005
Feed ME ^h^ (%)		ns	0.051	ns	ns	ns	ns
	Dairy shed	ns	ns	ns	ns	ns	ns
	Feed pads	ns	ns	ns	ns	ns	ns
	Holding areas	0.007	0.005	0.019	0.014	0.008	0.005
	Laneways	ns	ns	ns	ns	ns	ns
	Paddocks	ns	ns	ns	ns	ns	ns
	Yards	ns	0.036	ns	ns	ns	ns

^a^ F–prob estimates from residual maximum likelihood (REML) analysis, in which all effects were always positive. ^b^ Total land area visited by the lactating cows, which includes grazed paddocks, cropping, feeding areas such as feed pads, and sacrifice paddocks. ^c^ Total number of lactating, dry and springing cows averaged from data collected at five quarterly on-farm visits. ^d^ Cow numbers divided by the farm area. ^e^ Total litres of milk produced by the farm. ^f^ Calculated by dividing total production by the herd size for each farm. ^g^ Calculated by dividing total production by the farm area for each farm. ^h^ Feed metabolisable energy requirements (ME) as a percentage of total ME requirements that are imported onto farms. ns, not significant.

**Table 4:** Summary statistics for the total nutrient loads (kg herd^−1^) deposited over a 24 h period by lactating dairy cows on the 43 grazing system dairy farms.

	N	P	K	S	Ca	Mg
N	244	244	244	244	242	242
Minimum	2.3	0.4	2.1	0.2	0.3	0.3
Mean	112	15	85	11	21	13
Median	82	11	64	8	18	10
Maximum	776	114	502	72	104	51
Std Dev ^a^	99.74	13.64	70.77	10.04	17.20	9.66
CV ^b^ (%)	89	89	83	89	80	76
Skew	2.70	2.94	2.47	2.62	1.92	1.64
Kurt	10.76	13.94	9.35	9.83	4.64	3.02

^a^ Std. Dev, standard deviation, ^b^ CV, coefficient of variation.

**Table 5:** Calculated annual Nitrogen, Phosphorus, Potassium, Sulphur, Calcium, and Magnesium (t) excreted by the lactating herds, total annual nutrient imports and nutrients brought onto the farm in feed, in fertiliser or in both feed and fertiliser, for 41 grazing system conventional and organic dairy farms for which data were available.

	**Nitrogen (t)**					**Phosphorus (t)**				
	**Excreted ^a^**	**Annual Nutrient Imports ^b^**				**Excreted**	**Annual Nutrient Imports**			
		**Total**	**Feed**	**Fertiliser**	**Feed and Fertiliser**		**Total**	**Feed**	**Fertiliser**	**Feed and Fertiliser**
All farms ^c^ (*n* = 41)										
Minimum	6.79	8.79	1.18	0.00	1.45	1.11	0.41	0.27	0.00	0.39
Mean	34.40	55.06	21.36	23.56	44.92	4.86	8.54	3.55	4.49	8.03
Median	24.27	37.39	14.04	15.81	31.71	3.57	6.03	2.51	2.58	5.31
Maximum	154.92	245.16	167.04	154.31	212.68	21.10	52.40	26.52	25.11	51.62
Conventional farms (*n* = 37)										
Minimum	6.79	8.79	3.67	0.29	6.26	1.11	1.18	0.68	0.00	0.93
Mean	36.58	58.85	22.97	26.10	49.07	5.16	8.46	3.82	4.21	8.02
Median	26.92	41.58	16.28	16.79	34.15	3.69	5.96	2.59	1.94	5.28
Maximum	154.92	245.16	167.04	154.31	212.68	21.10	52.40	26.52	25.11	51.62
Organic farms (*n* = 4)										
Minimum	9.61	13.30	1.18	0.00	1.45	1.27	0.41	0.27	0.12	0.39
Mean	14.20	20.05	6.51	0.07	6.58	2.09	9.34	1.06	7.06	8.13
Median	13.15	18.91	7.15	0.01	7.15	1.85	10.70	0.91	8.61	10.20
Maximum	20.87	29.07	10.54	0.27	10.56	3.40	15.53	2.15	10.91	11.72
	**Potassium (t)**					**Sulphur (t)**				
	**Excreted**	**Annual Nutrient Imports**				**Excreted**	**Annual Nutrient Imports**			
		**Total**	**Feed**	**Fertiliser**	**Feed and Fertiliser**		**Total**	**Feed**	**Fertiliser**	**Feed and Fertiliser**
All farms ^c^ (*n* = 41)										
Minimum	5.07	2.94	1.02	0.00	2.33	0.68	0.46	0.34	0.00	0.34
Mean	26.12	22.53	11.89	8.98	20.87	3.46	8.35	2.16	4.10	6.26
Median	19.85	11.65	5.54	4.22	10.18	2.57	5.03	1.31	1.80	3.80
Maximum	94.89	154.39	103.70	64.38	146.98	14.48	61.03	22.33	25.99	48.33
Conventional farms (*n* = 37)										
Minimum	5.07	2.94	1.02	0.00	2.70	0.68	1.69	0.38	0.00	1.07
Mean	27.54	24.16	12.70	9.77	22.48	3.68	8.95	2.33	4.41	6.74
Median	20.43	12.15	5.70	4.50	11.58	2.76	5.61	1.40	1.82	3.83
Maximum	94.89	154.39	103.70	64.38	146.98	14.48	61.03	22.33	25.99	48.33
Organic farms (*n* = 4)										
Minimum	9.75	3.13	2.11	0.00	2.33	0.99	0.46	0.34	0.00	0.34
Mean	12.97	7.48	4.42	1.60	6.02	1.42	2.81	0.62	1.20	1.82
Median	11.55	6.59	3.46	0.73	5.92	1.25	2.81	0.60	0.76	1.49
Maximum	19.02	13.60	8.67	4.92	9.91	2.18	5.15	0.96	3.27	3.95
	**Calcium (t)**					**Magnesium (t)**				
	**Excreted**	**Annual Nutrient Imports**				**Excreted**	**Annual Nutrient Imports**			
		**Total**	**Feed**	**Fertiliser**	**Feed and Fertiliser**		**Total**	**Feed**	**Fertiliser**	**Feed and Fertiliser**
All farms ^c^ (*n* = 41)										
Minimum	1.38	2.09	0.53	0.00	0.89	1.10	0.77	0.24	0.00	0.24
Mean	6.58	40.09	4.62	24.74	29.36	3.87	7.64	2.25	1.05	3.31
Median	5.19	16.31	4.31	5.21	8.73	3.09	4.19	1.80	0.00	1.95
Maximum	19.32	301.25	26.47	195.44	196.79	11.23	69.79	11.81	22.60	25.98
Conventional farms (*n* = 37)										
Minimum	1.38	3.76	0.53	0.00	1.09	1.10	0.77	0.27	0.00	0.27
Mean	6.90	38.59	4.97	22.15	27.13	4.09	8.07	2.42	1.04	3.46
Median	5.67	13.58	4.57	4.61	8.72	3.27	4.51	1.93	0.00	2.09
Maximum	19.32	301.25	26.47	195.44	196.79	11.23	69.79	11.81	22.60	25.98
Organic farms (*n* = 4)										
Minimum	2.65	2.09	0.59	0.00	0.89	1.20	1.83	0.24	0.00	0.24
Mean	3.61	54.02	1.33	48.70	50.03	1.76	3.65	0.71	1.17	1.88
Median	3.60	52.96	1.39	44.88	46.80	1.60	3.50	0.77	0.00	0.91
Maximum	4.60	108.06	1.94	105.03	105.62	2.65	5.78	1.07	4.67	5.46

^a^ Excreted nutrients estimated based on total excreted in a day by the herd on each farm at each interview, multiplied by the number of days the farmer gave for that quarter, summed for the duration of the study and standardised for a 305-day lactation, ^b^ Annual total nutrients imported onto the farms or brought on in feed, fertiliser or both, based on data and samples collected over the year [4], ^c^ Data calculated for 41 grazing system farms; three farms had insufficient data. The summary statistics are shown for the conventional and the organic farms.

**Table 6:** Daily mean (median; min–max) excreted nutrients (kg) in the collected and recycled, not collected and recycled, or not collected and lost pools for 43 grazing system dairy farms.

	Collected	Not Collected	
Nutrient	Recycled ^a^	Recycled ^b^	Lost ^c^
N	7.4 (2.9; 0–101)	82 (60; 0–495)	9 (4.2; 0–168)
P	1 (0.38; 0–14.89)	11 (8; 0–72)	1.32 (0.57; 0–27.19)
K	5.5 (2.2; 0–68.7)	63 (49; 0–409)	6.3 (3.3; 0–109)
S	0.74 (0.29; 0–9.37)	8.3 (6.2; 0–56.6)	0.89 (0.45; 0–15.61)
Ca	1.34 (0.55; 0–23.13)	15.8 (12.8; 0–69.4)	0.78 (0.88; 0–31.3)
Mg	0.82 (0.32; 0–10.67)	9.1 (7.1; 0–43.2)	1.13 (0.55; 0–18)

^a^ Collected/recycled pool consisting of excreted nutrients deposited in dairy sheds, yards and on concreted feed pads where these are present. ^b^ Not collected/recycled pool consisting of nutrients excreted in paddocks. ^c^ Not collected/lost pool of excreted nutrients deposited on laneways, as well as holding areas and non-concreted feed pads where either or both of these are present.

**Table 7:** Daily mean (median; min-max) nutrients (kg) in the collected and recycled pool, and in the not collected and lost pool for the seventeen farms (i) where nutrients deposited in excreta to feed pads and holding areas were not collected, or (ii) if these deposited nutrients were collected.

	Feed Pads and Holding Areas (i)		Feed Pads and Holding Areas (ii)	
	Collected and Recycled ^a^	Not Collected and Lost ^b^	Collected and Recycled	Not Collected and Lost
N	6.3 (2.7; 0–82.5)	9 (4; 0–103)	7.9 (2.7; 0–103)	6.7 (4.5; 0–34)
P	0.90 (0.36; 0–13.15)	1.45 (0.58; 0–27.19)	1.23 (0.37; 0–27.19)	0.97 (0.68; 0–6.78)
K	4.7 (1.9; 0–64.7)	6.5 (3.1; 0–75)	5.7 (1.9; 0–75)	5.1 (3.5; 0–27.3)
S	0.6 (0.23; 0–7.23)	0.91 (0.4; 0–12.93)	0.78 (0.25; 0–12.93)	0.64 (0.47; 0–3.23)
Ca	1.17 (0.51; 0–15.26)	1.82 (0.86; 0–31.3)	1.56 (0.52; 0–31.3)	1.23 (0.91; 0–5.95)
Mg	0.75 (0.33; 0–8.75)	1.22 (0.55; 0–18.04)	1.03 (0.33; 0–18.04)	0.80 (0.66; 0–2.91)

^a^ Excreted nutrients collected in dairy sheds, yards, and concreted feed pads where these are present (i), and compared to recycled nutrients if excreta deposited in non-concreted feed pads and holding areas were collected; (ii). ^b^ Excreted nutrients deposited on laneways, as well as holding areas and non-concreted feed pads where either or both of these are present (i), and compared to nutrients lost only in laneways (i.e., all excreta collected from feed pads and holding areas) (ii).

**Table 8:** Summary statistics (mean, median, range and semi–quartile range) for nutrient loads (kg) excreted over a lactation (305 days) by the lactating herds at the dairy shed and yards, within 100 m of the dairy shed, and in paddocks grazed overnight or in the daytime, on 42 dairy farms.

		Mean	Median	Min–Max	SQR
Nitrogen (kg)					
	Dairy shed ^a^	1882	926.4	81.8–21,444	855.5
	<100 m ^b^	3917	1589	0–19,802	1575.7
	Overnight ^c^	14,693	10,401	2937–60,330	5351
	Daytime ^d^	10,061	8140	791.2–49,818	2548
Phosphorus (kg)					
	Dairy shed	262	131.3	13.98–2877	115.275
	<100 m	625.2	222.6	0–4035	251.08
	Overnight	2049	1405	440.4–7467	577
	Daytime	1392	993.3	137–6720	353.8
Potassium (kg)					
	Dairy shed	1411	773.7	60.47–13,085	675.4
	<100 m	2624	1178	0–13,798	1044.25
	Overnight	11,493	8712	1653–48,714	3686.5
	Daytime	7713	5690	588.6–30,235	2114
Sulphur (kg)					
	Dairy shed	188.5	93.48	7.967–2011	87.265
	<100 m	379	145	0–2478	167.49
	Overnight	1496	1051	285.8–6448	507.85
	Daytime	1008	744.1	78.23–4661	266.25
Calcium (kg)					
	Dairy shed	345.1	209.6	20.3–2760	149.56
	<100 m	717.6	308.3	0–2970	386.35
	Overnight	2840	2317	610.8–9740	1009.5
	Daytime	1897	1419	326.5–6258	563
Magnesium (kg)					
	Dairy shed	207.3	117.8	13.57–1490	100.465
	<100 m	501.7	221.4	0–2621	208.465
	Overnight	1632	1131	461.2–5888	513.05
	Daytime	1106	812.4	129.8–3648	238.25

^a^ Dairy shed and yards in which cows spend time during milking, generally twice daily; *n* = 478 (N, P, K, S), 474 (Ca, Mg). ^b^ Nutrients deposited in feed pads and holding areas, both on average less than 100 m from the dairy shed; *n* = 160 (N, P, K, S), 158 (Ca, Mg). ^c^ ‘Overnight’ paddocks were designated as those paddocks in which the cows were placed after the last milking in the evening and from which the cows were collected before the first milking in the morning. The ‘overnight’ paddocks were on average 118 m closer to the dairy shed than the ‘daytime’ paddocks; *n* = 239 (N, P, K, S), 237 (Ca, Mg). ^d^ ‘Daytime’ paddocks designated as those paddocks grazed by the lactating herds during the day, typically between the morning and evening milking. These paddocks were a mean distance of 742 m from the dairy shed; *n* = 239 (N, P, K, S), 237 (Ca, Mg).

**Table 9:** Summary statistics (mean, median, range) for nutrient loading rate (kg ha^−1^) excreted over a lactation (305 days) by the lactating herds in ‘overnight’ and ‘daytime’ paddocks, as well as for nutrients applied as fertilisers, on 42 grazing system dairy farms.

		Mean	Median	Min–Max
Nitrogen (kg ha^−1^)				
	Overnight ^a^	200.8	145.9	9.7–607.9
	Daytime ^b^	135.5	113.6	15.5–308.4
	Fertiliser ^c^	141.3	116.0	0–429
Phosphorus (kg ha^−1^)				
	Overnight	28.06	23.98	2.1–119.5
	Daytime	18.61	16.1	2.5–45
	Fertiliser	23.99	18.03	0–90.8
Potassium (kg ha^−1^)				
	Overnight	155.8	145.5	8.6–400.7
	Daytime	103.9	105	11.7–210.9
	Fertiliser	42.8	31.5	0–226.9
Sulphur (kg ha^−1^)				
	Overnight	20.56	15.2	1.1–67.8
	Daytime	13.69	11.98	1.5–30.5
	Fertiliser	21.48	16.98	0–89.0
Calcium (kg ha^−1^)				
	Overnight	38.98	34.27	1.8–93.2
	Daytime	26.07	24.06	1.8–67.3
	Fertiliser	158.15	47.04	0–1313
Magnesium (kg ha^−1^)				
	Overnight	22.99	18.73	1.7–70
	Daytime	15.41	14.39	1.6–37.4
	Fertiliser	8.08	0.00	0–178

^a^ ‘Overnight’ paddocks were designated as those paddocks in which the cows were placed after the last milking in the evening and from which the cows were collected before the first milking in the morning. The ‘overnight’ paddocks were on average 118 m closer to the dairy shed than the ‘day’ paddocks [16]. ^b^ ‘Daytime’ paddocks designated as those paddocks grazed by the lactating herds during the day, typically between the morning and evening milking. These paddocks were a mean distance of 742 m from the dairy shed. ^c^ Application rates of fertilisers are based on total fertiliser nutrients applied to the farm divided by the total area of all pasture paddocks on each farm.

## Data Availability

Datasets are available upon request from the submitting author.

## Supplementary Materials

The following supporting information can be downloaded at: https://www.mdpi.com/article/10.3390/ani13081404/s1. Table S1: Number of daily records for each farm based on the times farms were interviewed and number of herds present on each interview date, as well as the number of records for the six management units visited by each herd on each farm on each interview date.
