# Supplementary material for: Estimating Excreted Nutrients to Improve Nutrient Management for Grazing System Dairy Farms
Source: Animals (Basel). 2023 Apr 19;13(8):1404. doi: 10.3390/ani13081404 (PMC10135339; doi:10.3390/ani13081404)
Supplement: Supplementary file 1 [file animals-13-01404-s001.zip › animals-2326412-supplementary.pdf]

Table S1 Number of daily records for each farm based on the times farms were interviewed and number of herds present on each interview date, as well as the number of records for the six management units visited by each herd on each farm on each interview date.

Farm		Number of	Number of records	
Number	Herds	Times interviewed	Daily	Management Units
1	2	4	8	48
1	3	1	3	18
43	1	3	3	18
43	2	2	4	24
37	2	2	4	24
37	1	3	3	18
36	1	2	2	12
36	2	3	6	36
33	1	4	4	24
33	2	1	2	12
30	1	4	4	24
29	2	5	10	60
26	1	4	4	24
23	1	3	3	18
23	2	2	4	24
19	1	4	4	24
19	2	1	2	12
18	1	4	4	24
18	2	1	2	12
16	1	3	3	18
16	2	1	2	12
12	1	4	4	24
6	1	3	3	18
6	2	2	4	24
3	2	4	8	48
3	1	1	1	6
2	1	2	2	12
2	2	3	6	36
All other [a]	1	5	135	810
			244	1464

[a] All other study farms (Farms 4-5, 7-11, 13-15, 17, 20-22, 24-25, 27-28, 31-32, 34-35, 38-42) had one herd present each of the 5 farm visits. The farm numbers in the table were all visited 5 times except for Farms 12,16, 26, 30 (visited 4 times).
